# Increased EEG gamma power under exposure to drug-related cues: a translational index for cue-elicited craving in METH-dependent individuals

**DOI:** 10.1186/s12888-023-04892-9

**Published:** 2023-05-25

**Authors:** Dong-xu Li, Xiang-yi Zhou, Qian-qian Lin, Yue Wu, Cheng Hu, Zhi-hua Shen, Yong-guang Wang

**Affiliations:** 1 School of Mental Health and Psychological Sciences, Anhui Medical University, Hefei, Anhui Province China; 2 Affiliated Mental Health Center, Hangzhou Seventh People’s Hospital, 305 Tianmushan Road, Hangzhou, Zhejiang Province China; 3 The Fifth Hospital of Ruian, Ruian, Zhejiang Province China; 4 Shiliping Compulsory Rehabilitation Center, Zhejiang, China; 5 Zhejiang Provincial Institute of Drug Abuse Research, Hangzhou, Zhejiang Province China

**Keywords:** Methamphetamine, Craving, Virtual reality, Counterconditioning, Gamma oscillations

## Abstract

**Background:**

This study explored the feasibility of using EEG gamma-band (30–49 Hz) power as an index of cue-elicited craving in METH-dependent individuals.

**Methods:**

Twenty-nine participants dependent on methamphetamine (METH) and 30 healthy participants were instructed to experience a METH-related virtual reality (VR) social environment.

**Results:**

Individuals with METH dependence showed significantly stronger self-reported craving and higher gamma power in a VR environment than healthy individuals. In the METH group, the VR environment elicited a significant increase in gamma power compared with the resting state. The METH group then received a VR counterconditioning procedure (VRCP), which was deemed useful in suppressing cue-induced reactivity. After VRCP, participants showed significantly lower self-reported craving scores and gamma power when exposed to drug-related cues than the first time.

**Conclusions:**

These findings suggest that the EEG gamma-band power may be a marker of cue-induced reactivity in patients with METH dependence.

## Introduction

Methamphetamine (METH) dependence is a crucial public health concern. In the past 10 years, the number of illicit METH users in China has rapidly increased [1]. For drug users, one significant symptom is the craving elicited by drug-related cues [2]. Exposure to such cues may trigger drug-seeking and drug-taking behaviors, which could influence relapse [3–7]. Thus, improving our understanding of cue-induced cravings and developing effective interventions are necessary.

Over the past few decades, neuroimaging techniques have been significant in cue reactivity research. Functional magnetic resonance imaging (fMRI) studies have revealed several processes associated with cue-induced cravings [8, 9]. For example, the central executive network, which is linked with cognitive control and executive functions, is engaged during appetitive cue reactivity [8]. Reward and emotional processing could also influence cue-induced craving [8, 10, 11].

Electroencephalogram (EEG) studies have revealed the brain-mind relationship in cue-induced reactivities based on oscillatory brain dynamics. Previous studies have reported increased activity of beta oscillations due to alcohol or cocaine dependence [12–14]. Heroin-dependent individuals have enhanced beta and gamma powers than healthy controls (HCs) [15]. Nicotine administration elicits EEG activity shifts from low (delta, theta, alpha-1) to high (alpha-2, beta) frequencies, indicating a state of arousal [16–18]. Some researchers found that METH-dependent participants showed lower delta and alpha powers with higher beta and gamma powers compared to healthy participants [19]. Zhao et al.’s study of METH use disorder revealed that a cue-induced craving was higher after abstinence of 1–3 months, along with a decreased power spectrum for theta and alpha, and an increase in the beta frequency band [20]. In contrast, some evidence suggests that gamma activity in the Medial Prefrontal Cortex/Orbital Frontal Cortex and right Dorsolateral Prefrontal Cortex decreases after cue exposure [5].

Thus, EEG is an objective and precise method for detecting brain dysfunction in people with substance use disorder, and EEG abnormalities may reflect the underlying changes in brain function caused by chronic drug abuse. In most studies, cue-induced cravings were correlated with increased power in the beta frequency band, which was correlated with perceptual and motor processes [14, 19, 20]. However, only a few studies have investigated the correlation between cue-induced cravings and gamma oscillations. Some studies reported that low-frequency EEG signals are related to alertness and motor imagery, whereas high-frequency signals are related to memory, attention, and emotion [21–25]. Thus, such high-frequency oscillations are not specific to a particular cognitive function, but represent global binding across various neurons and networks [26–29].

Evidence from previous studies suggested that gamma oscillations represent activation-related markers of neural communication and are important in complex multisensory perceptual paradigms (e.g., watching a video) as well as in high-order cognitive processes such as attention, memory, and emotion [26, 28, 30–34]. Since these functions influence cue-induced craving, extracting the features of gamma activity might help us understand how the brains of METH abusers react to drug-related cues.

In our previous study, we developed a METH-related virtual reality (VR) social environment and a VR counterconditioning procedure (VRCP) for cue-induced craving assessment and treatment. Patients with METH dependence showed a significant increase in craving scores when exposed to METH-related cues in the VR environment, with a corresponding increase in heart rate variability. However, participants who received VRCP showed a larger decrease in the craving score from baseline to follow-up assessments compared to those who did not receive VRCP. The current study explores the sensitivity of EEG gamma-band power as an index of cue-induced craving in METH-dependent individuals when they are exposed to a METH-related VR environment. Specifically, we recorded the EEG signals of participants under different conditions (cue-induced vs. rest) before and after receiving VRCP.

Based on previous studies [35], we hypothesized that (1) the VR environment might elicit larger gamma activity in the METH group than in HCs and (2) receiving VRCP might lead to a decrease in gamma frequency power in METH-dependent individuals during cue-induced conditions, compared to the data recorded before VRCP. Two studies were designed to test these hypotheses.

## Methods

### Participants

Power analyses were conducted with G-Power 3.1.9.3 (effect size = 0.6; means: difference from constant; α < 0.05; 1-β > 0.8).

METH-dependent participants were recruited from the Compulsory Rehabilitation Center in Shiliping, Zhejiang Province, China. Patients admitted to the rehabilitation center first underwent 2 weeks of detoxification. Thereafter, psychologists decided whether or not they would receive psychological treatment. Psychological treatment includes CBT and supportive psychotherapy. In this study, participants did not receive any other treatment except for VRCP before and throughout the testing period. First, they underwent screening, including demographic information, drug use history, and psychiatric evaluation. All participants were evaluated using psychopathological questionnaires including the Self-Rating Anxiety Scale (SAS), Self-Rating Depression Scale (SDS), and Barratt Impulsiveness Scale version 11 (BIS-11). The necessary inclusion criteria were the diagnosis of METH dependence based on the DSM-IV, as determined by an experienced psychiatrist. Exclusion criteria were: (1) taking medications at the time of the study, (2) other concurrent neuropsychiatric diseases that might induce EEG alteration, and (3) other psychoactive substance usage history. In total, 35 male participants with METH dependence were enrolled in this study, 6 of whom quit compulsory intervention before completing the study procedure. Finally, 29 participants with METH dependence completed all measurements.

In addition, 30 healthy male participants were recruited from the local community through advertisements. None had a history of drug use or any neurological/psychiatric disorder or major physical disease.

### Methods

#### Assessment procedure

**Fig. 1:**
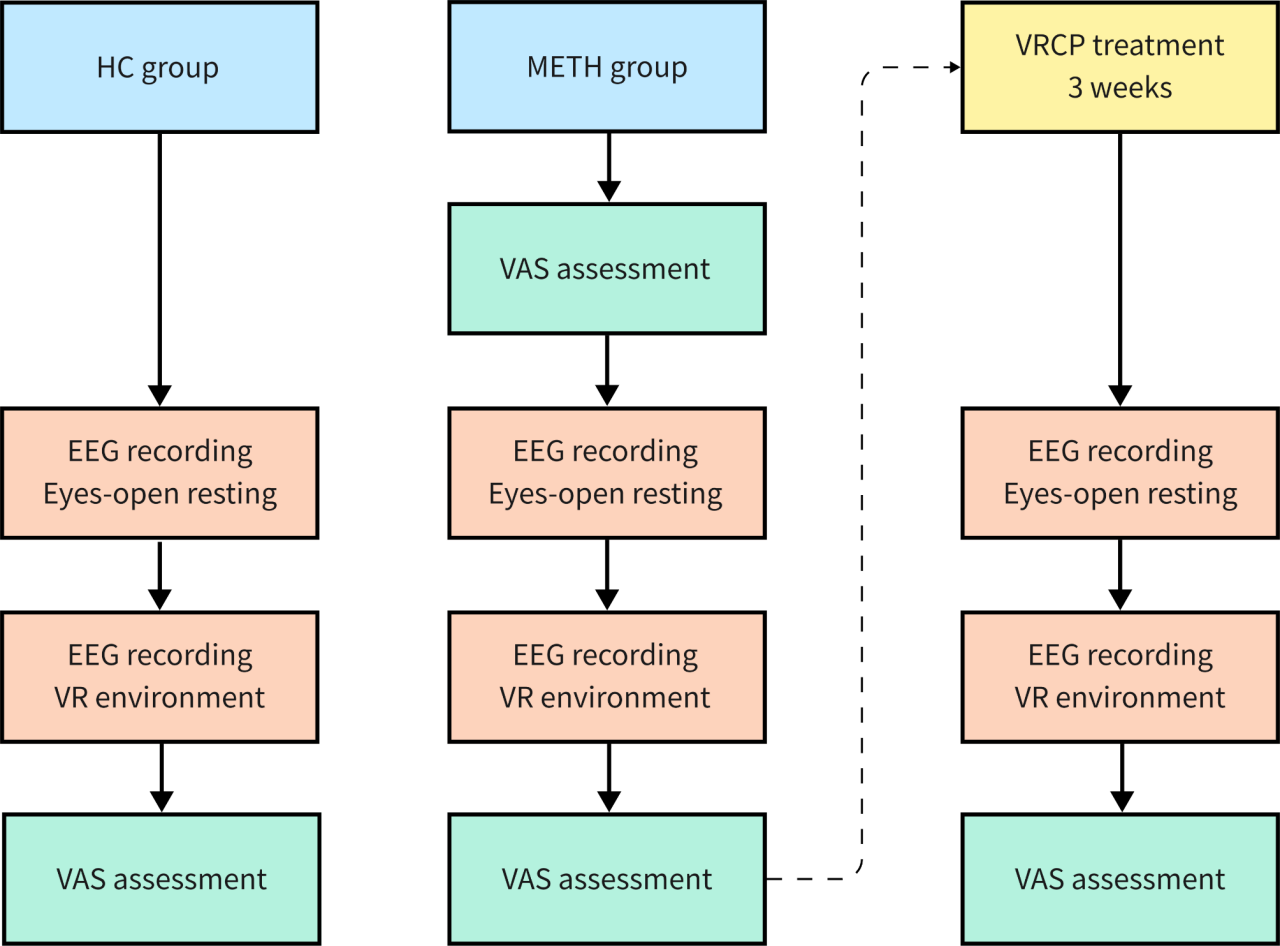
Flow chart of present work

Figure 1 is a schematic diagram of the assessment procedure. First, participants were seated in a quiet room for EEG recording, which included a six-minute eyes-open resting-state EEG. They were then introduced to the VR device (i.e., VR helmets and headphones). After participants adapted to the VR scene, they were instructed to watch an eight-minute VR video that simulates a real METH-related social context including various METH-related cues, and the concurrent EEG signals were recorded (i.e., cue-induced EEG). Before the EEG recording and immediately after the VR video, participants in the METH group were asked to answer three questions on a visual analog scale (VAS) (i.e., VAS-craving, VAS-liking, and VAS-using). For the HCs, these questions were asked only after the VR video.

After the assessment, participants in the METH group were instructed to receive six sessions of VRCP treatment. In each session, participants were required to watch one VR video following a VRCP (VR treatment system, Hangzhou Seventh Science and Technology Co., Ltd.). The VRCP sessions were run twice a week. After completing all six sessions of treatment, resting-state and cue-induced EEGs were recorded with the same settings as mentioned above. The VAS assessment was performed only after the cue video.

#### EEG recording

The EEG data were recorded using a 32-channel system with a sampling rate of 2048 Hz, and the electrode placement followed the international 10–20 system (eegoTM mylab, ANT Neuro, The Netherlands). The reference electrode was placed on the CPz, and all channel impedances were kept below 10 kΩ.

#### VR videos

The METH-cue VR video comprised several drug-related social scenes including visual, auditory, and action cues and social information such as facial expressions and social interactions. Preliminary work has indicated that this virtual social environment could efficiently elicit reliable craving.

In addition, six VR videos were used in the VRCP, and each video lasted approximately five minutes. All these videos had similar beginnings, depicting a story of men/women using METH together. The participants then viewed the characters in the videos to experience different adverse consequences caused by using METH. Preliminary work has indicated that this treatment procedure is useful for suppressing cue-induced reactivity in patients with METH dependence. More details regarding the METH-cue VR video and VRCP can be found in our previous study.

#### VAS assessments

Participants were asked to answer three questions on a VAS by choosing the most suitable option for each question. The first question was regarding METH-craving: “How much do you crave METH/ice right now?” (ranging from 0 to 10, with 0 indicating “no craving at all” and 10 indicating “extremely strong craving”). The second question enquired about METH usage: “If you have access to METH/ice right now, how likely would you be to use it?” (ranging from 0 to 10, with 0 indicating “certainly not” and 10 indicating “certainly”). The third question was: “To what extent do you find METH/ice pleasant/unpleasant?” (ranging from 0 to 10, with 0 indicating “very unpleasant,” 5 indicating “neither unpleasant nor pleasant,” and 10 indicating “very pleasant”).

### EEG data preprocessing and analysis

All EEG data were processed using MATLAB (MathWorks, Natick, MA, USA). Preprocessing procedures were performed to exclude artifacts. EEG data were bandpass filtered between 0.1 and 70 Hz, with the notch frequency set to 50 Hz. The EEG data were subsequently down-sampled to 200 Hz and re-referenced to the mastoid electrodes (i.e., average M1–M2). An independent component analysis was performed to decompose the EEG signals. Components containing artifacts of eye/muscle movements were rejected. Then, the EEG was segmented into 2-second epochs. Epochs with excessive motor activity were removed if necessary.

A spectral analysis was performed using the EEGLAB plugin (Darbeilial, https://github.com/embar-/eeglab_darbeliai/wiki/0.%20EN). The window length and frequency resolution were set to two seconds and 0.1 Hz, respectively. Thus, for each 2-s segment of the EEG, Power Spectral Density data were obtained and then averaged. For statistical purposes, scalp electrodes were divided into nine clusters comprising one or two electrodes: Anterior Left (F3–F7), Anterior Central (Fz), Anterior Right (F4–F8), Central Left (T7–FC5), Central Central (Cz), Central Right (T8–FC6), Posterior Left (P7–CP5), Posterior Central (Pz), and Posterior Right (P8–CP6). The clustering resulted in a 3 × 3 grid along a two-dimensions-defined region (anterior-central-posterior) and hemisphere (left-central-right). Spectrum power (dB) in the gamma range (30–49 Hz) was computed separately for each cluster (for clusters comprising two electrodes, the power was averaged).

### Statistical data analysis

A two-sample t-test and paired t-test were conducted to compare age and VAS score between groups and between conditions, respectively. Gamma activity was analyzed using four-way repeated-measures ANOVAs. For the contrasts between the METH and HC groups, the within-subject factors considered were Condition (rest/cue-induced) and Region and Hemisphere. Group was considered the between-subject factor. We conducted another rmANOVA to compare the data recorded before and after VRCP (METH group only). The Condition, Region, Hemisphere, and Intervention (before VRCP/after VRCP) were considered as within-subject factors. Bonferroni corrections were used for the multiple comparisons.

**Table 1:** Demographic characteristics and VAS scores

Study 1	METH *(n = 29)*	HC *(n = 30)*	t-value	p-value
Age (years)	32.93 ± 5.64	29.90 ± 6.713	1.874	0.066
Years of METH use	9.69 ± 4.02			
Days of abstinence	323.66 ± 134.40			
SAS	31.41 ± 6.10	27.93 ± 5.73	2.259	0.028
SDS	35.62 ± 9.26	28.63 ± 6.63	3.341	0.001
BIS-11	57.83 ± 9.60	50.87 ± 11.02	2.584	0.012
**Resting-state VAS**				
METH-craving	2.41 ± 1.61			
METH-using	5.34 ± 3.50			
METH-liking	3.93 ± 2.25			
**Cue-induced VAS (before VRCP)**				
METH-craving	4.76 ± 2.32	0.17 ± 0.65	10.41	< 0.001
METH-using	7.00 ± 3.18	0.30 ± 1.15	10.82	< 0.001
METH-liking	4.76 ± 1.90	0.93 ± 1.78	7.98	< 0.001
**Cue-induced VAS (after VRCP)**				
METH-craving	1.07 ± 1.36			
METH-using	2.83 ± 3.11			
METH-liking	2.66 ± 2.27			

## Results

### Demographic characteristics, psychopathological assessment, and VAS

**Fig. 2:**
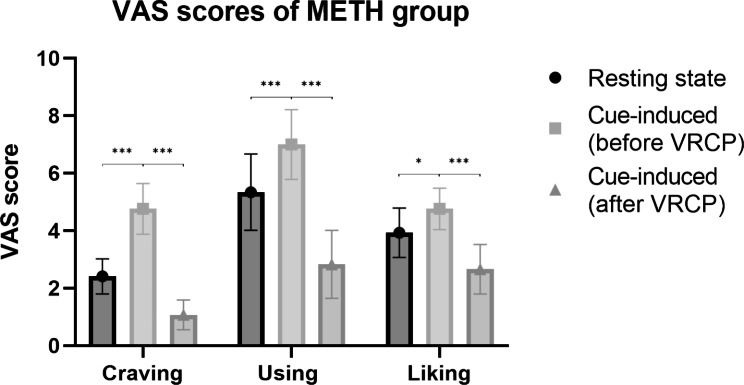
VAS scores of METH group in different conditions

No significant differences in age were observed between the METH and HC groups. Figure 2 shows the VAS scores of the METH group under different conditions. In the METH group, watching cue-related VR videos led to a significant increase in VAS-craving (t = 7.022, P < 0.001), VAS-craving (t = 4.261, P < 0.001), and VAS-liking (t = 3.041, P = 0.005). The METH group reported significantly higher VAS scores for METH-craving (t = 10.412, P < 0.001), METH-using (t = 10.820, P < 0.001), and METH-liking (t = 7.979, P < 0.001) compared to the HC group. After VRCP, the METH group reported a significant decrease in VAS-craving (t = 8.943, P < 0.001), VAS-using (t = 7.609, P < 0.001), and VAS-liking (t = 5.549, P < 0.001). Participants’ demographic characteristics and VAS results are presented in Table 1.

### Gamma power

**Fig. 3:**
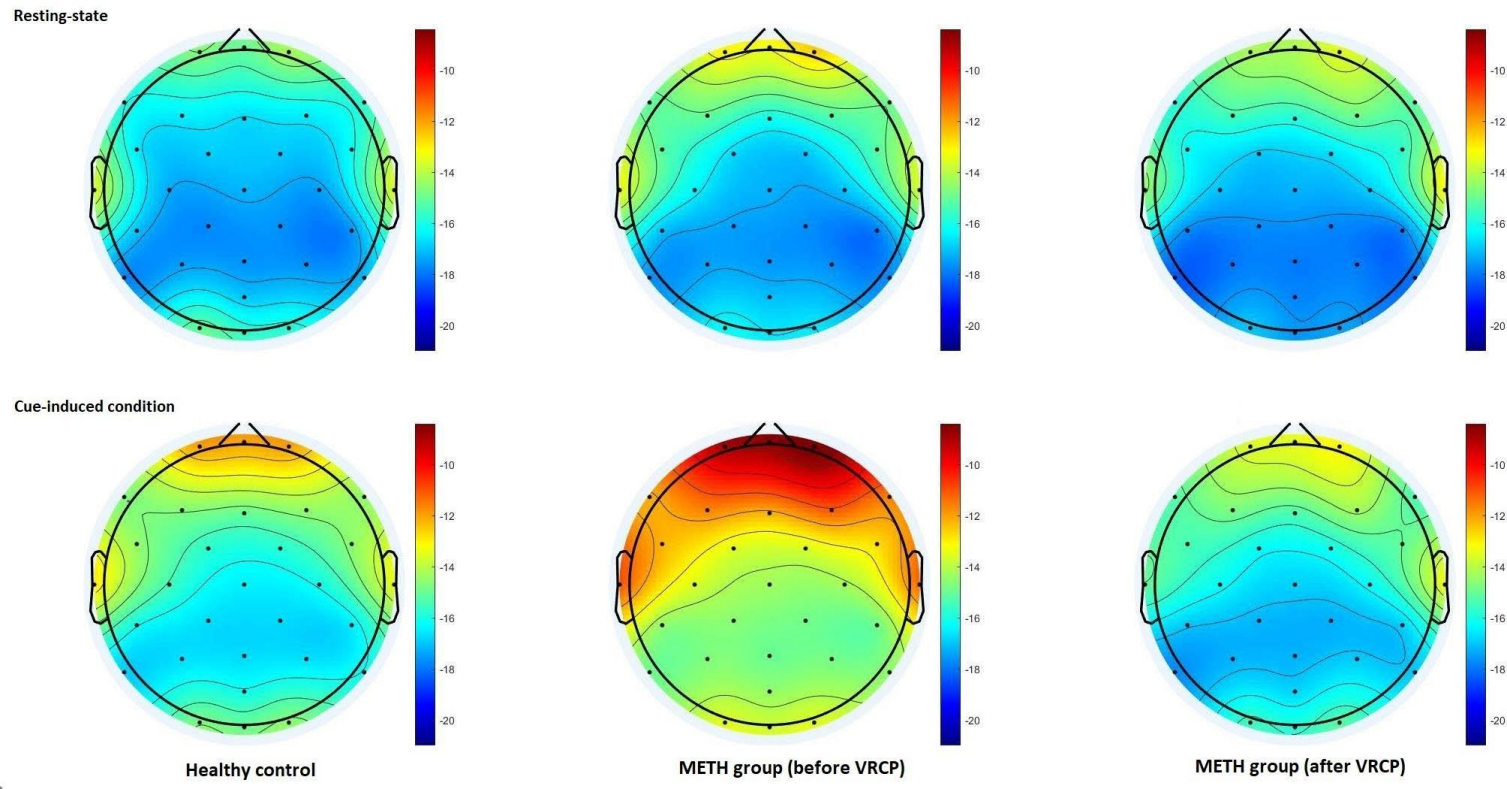
Topographic map of gamma power (dB), for each group and condition

Figure 3 presents a topographic map of the gamma power for each group and condition. The first ANOVA revealed a significant main effect of Condition (F = 24.637, P < 0.001), Region (F = 72.088, P < 0.001), Hemisphere (F = 26.475, P < 0.001), and Group (F = 6.236, P = 0.015). There were significant interactions between Condition and Group (F = 4.500, P = 0.038), Region and Group (F = 4.241, P = 0.023), Condition and Region (F = 6.902, P = 0.003), and Region and Hemisphere (F = 33.765, P < 0.001). Further analyses of the Condition × Group effect revealed that compared with the HC group, the VR video elicited larger gamma activity in the METH group (P = 0.001). Analyses of the Region × Condition effect showed that compared with the resting state, the VR environment elicited larger gamma activity in scalp regions (All Ps < 0.001). Regarding the Region × Group effect, participants in the METH group had larger gamma activity in the anterior (P = 0.001) and central (P = 0.043) regions compared with HCs.

**Fig. 4:**
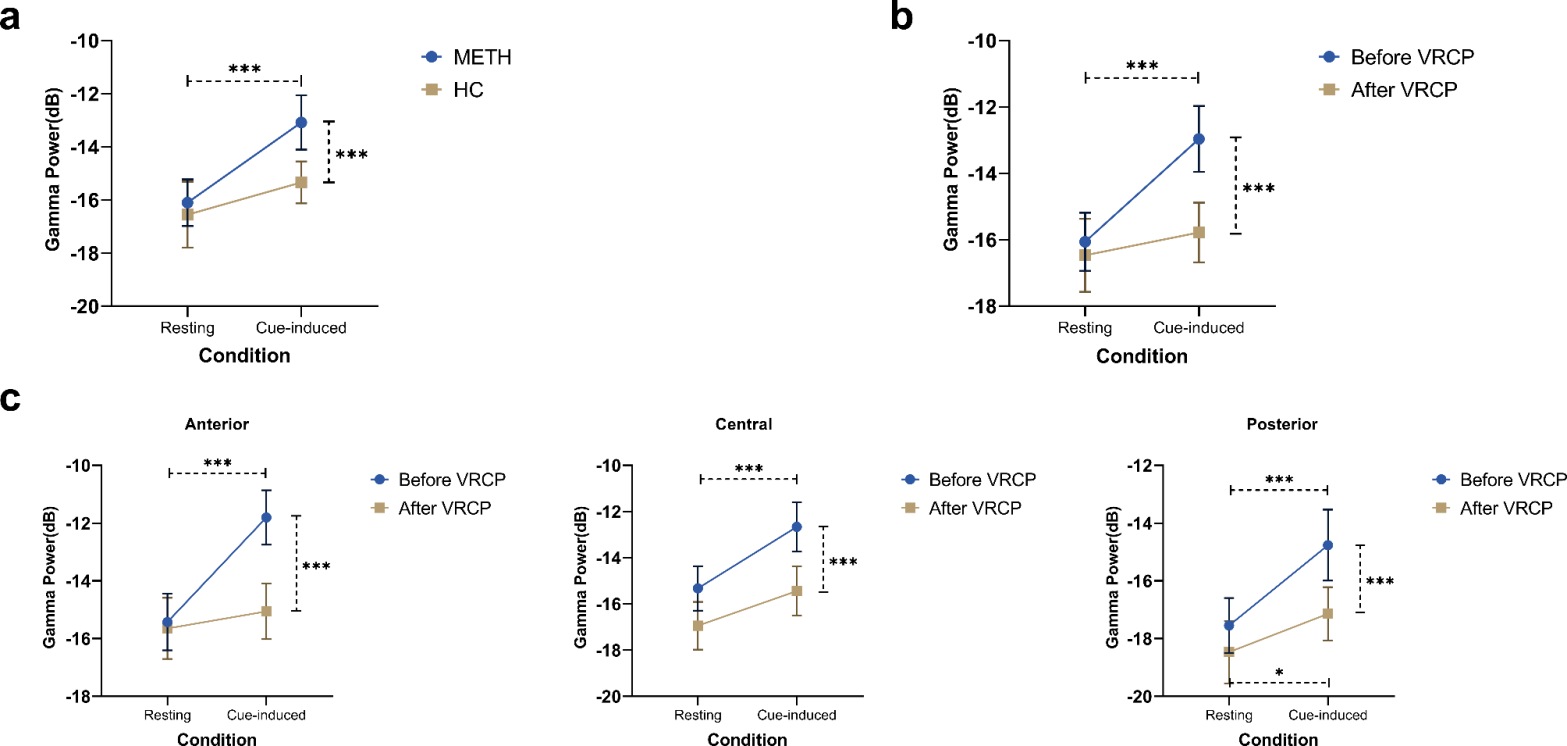
Results of gamma power. (**a**) Effect of drug-related VR environments on gamma power in METH and HC groups. (**b**) Effect of VRCP treatment on global gamma power in METH group. (**c**) Effect of VRCP treatment on gamma power across different scalp regions in METH group. Error bar: 95% CI; ***: p < 0.001, *: p < 0.05

The second ANOVA revealed a significant main effect of Intervention (F = 19.626, P < 0.001), Condition (F = 17.611, P < 0.001), Region (F = 87.843, P < 0.001), and Hemisphere (F = 15.144, P < 0.001). Significant interactions were found between Intervention and Condition (F = 21.179, P < 0.001), Region and Hemisphere (F = 25.295, P < 0.001), and significant Intervention × Condition × Region effects (F = 5.563, P = 0.011). Further analyses revealed that after receiving VRCP, METH-dependent individuals showed significantly lower gamma power when they were exposed to drug-related cues compared with the data recorded before VRCP (all Ps < 0.001). The drug-related VR videos no longer elicited a significant increase in gamma power in the anterior and central areas, but still activated gamma activity in the posterior area (P = 0.033). The results of the post-hoc analysis are presented in Fig. 4 for each region.

## Discussion

Our study aimed to evaluate how craving elicited by a METH-related VR environment modulates EEG activity in METH abusers. Previous studies suggested that gamma oscillations influence complex multisensory perceptual paradigms [28]; however, no study has investigated gamma power using the METH-related VR environment. In this study, we first measured the EEG spectrum power of METH users under different conditions: resting state and exposure to a METH-related VR environment. The VR environment elicited a significantly larger increase in gamma power in the METH group than in the HC group. This result supports our first hypothesis that EEG gamma power may represent a marker of cue-induced reactivity in METH-dependent disorders. In addition, Fig. 4 shows that gamma activity provoked by drug-related cues was mainly recorded by the frontal electrodes. This finding may suggest some spatial information of the craving process; however, the results should be interpreted with caution [36].

Previous studies emphasized that gamma-band oscillations are significant in object representation [37]. Subsequent studies demonstrated that gamma-band activity is related to bottom-up representation and top-down processes. Gamma-band responses are closely associated with selective attention [38]. Given the relationship between gamma activity and top-down representation, the attractive stimuli exhibited by VR may strongly activate the top-down processes of patients with METH dependence, enhancing their gamma-band activity. Aligning with our results, some authors have concluded that frontal gamma activity is linked to top-down processes [39, 40].

Furthermore, drug-related reactivity impacts emotional properties. The processing of rewards includes the components of “liking” and “wanting” [41–43]. “Liking” refers to the hedonic impact or affective process within pleasure. Based on these models, the increase in METH-liking scores after watching VR videos represents a growing positive affect. Consistent with our previous study [35], our results showed that patients reported a significant decrease in the VAS score for METH-liking after receiving VRCP, which suggests that VRCP attenuated the affective valance of METH cues.

The power spectrum analysis after VRCP treatment revealed a similar trend in the gamma power. Our results showed that VRCP treatment decreased gamma power when participants were exposed to a METH-related VR environment, compared with their condition before VRCP. The association between gamma oscillations and emotional processing may explain this. An increase in the spectral power of the gamma range has been reported in positive emotional tasks [44]. Some studies have shown increased gamma activity in the right hemisphere for emotional pictures (pleasant and unpleasant) compared to neutral pictures [31, 45], although our results did not show right hemisphere dominance. Maffei et al. found that watching emotional movies elicited greater gamma activity than watching neutral clips [28].

Likewise, evidence for the possible role of gamma-band activity in learning and short-term memory has been reviewed [29, 46]. Some studies have demonstrated that fast oscillations reflect the synchronous activity of large ensembles of neurons [30]. A reasonable explanation is that such high-frequency oscillations are not specific to a particular cognitive process, but rather represent the functional communication between various neurons and networks [26–29]. Together, both these processes could lead to the observed effects of gamma oscillation.

However, this study has some limitations. First, this study enrolled only male participants, and the sample size was small, making it difficult to generalize the results. Although no statistically significant difference was found in age between the two groups, the difference should not be ignored in terms of brain functioning. Future studies should investigate female METH dependence patients too and expand the scale. Second, no source analysis was performed because of the constraints of the EEG system. Therefore, the brain networks underlying the observed effects remain unclear. Third, VR devices and environments may disturb participants’ baseline condition. To rule out such bias, the control condition should be set up in a VR environment. Fourth, the control group did not respond to the VAS at the resting state. Even if they are not users, they may have relevant knowledge or experiences. Furthermore, participants were not evaluated with a more comprehensive craving questionnaire, leading to a lack of comprehensive information about their craving. In addition, abstinence may induce incubation of a cue-induced drug craving. A previous study [20] reported that a cue-induced craving of METH was higher after 1–3 months’ abstinence than after 3–6, 6–12, and 12–24 months. This effect on a cue-induced craving may impact the different responses to METH-related cues, and should be considered in future studies. Last and most important, the present study did not set up a control condition for VRCP. The observed changes in VAS and gamma-band power after VRCP might be related to other potential variables such as habituation to the stimuli presented in the videos, rather than be an effect of VRCP.

In conclusion, the present study found some preliminary results, suggesting that gamma power may be a marker of cue-induced reactivity in METH-dependent disorders.

## Data Availability

The datasets generated during and/or analyzed during the current study are available from the corresponding author on reasonable request.
